# Reliability and validity of depression anxiety stress scale (DASS)-21 in screening for common mental disorders among postpartum women in Malawi

**DOI:** 10.1186/s12888-022-03994-0

**Published:** 2022-05-24

**Authors:** Ernest Moya, Leila M. Larson, Robert C. Stewart, Jane Fisher, Martin N. Mwangi, Kamija S. Phiri

**Affiliations:** 1 Training and Research Unit of Excellence (TRUE), 1 Kufa Road, PO Box 30538, Chichiri, BT3, Blantyre, Malawi; 2 Department of Public Health, School of Global and Public Health, Kamuzu University of Health Sciences, Private Bag 360, Chichiri, BT3, Blantyre, Malawi; 3 Department of Health Promotion, Education, and Behavior, Arnold School of Public Health, University of South Caroline, 915 Green Street Columbia, SC 29208 USA; 4 Division of Psychiatry, University of Edinburgh, Kennedy Tower, Royal Edinburgh Hospital, Morningside Park, Edinburgh, UK; 5 Global and Women’s Health Unit, School of Public Health and Preventive Medicine, Monash University, Melbourne, VIC Australia

**Keywords:** Anxiety, Depression, Internal reliability, Validation, Sensitivity, Specificity, Postpartum, Women, Malawi, DASS-21

## Abstract

**Background:**

Approximately one in five women who have recently given birth suffer from common mental disorder (CMD), particularly depression and/or anxiety. Most available CMD screening tools in most low- and middle-income countries do not screen for more than one mental health problem. Having a screening tool that is free to use, short in assessment time, and used to screen for more than one CMD is appealing in a resource-constrained setting.

**Method:**

We conducted a criterion validation study of the Chichewa translated and adapted DASS-21 instrument against gold standard diagnoses of depression and anxiety disorders using an independently administered Structured Clinical Interview for DSM-IV (SCID). We compared the performance of the DASS-depression subscale with the Edinburgh Postpartum Depression Scale (EPDS). Internal reliability was reported using both Cronbach’s alpha and ordinal alpha. The DASS-21 and EPDS ability to discriminate cases from non-cases was assessed by receiver operating characteristics (ROC) analysis. We selected cut-off points for DASS-21 and EPDS that maximise both sensitivity and specificity.

**Results:**

One hundred fifteen participants were administered all the measures. Approximately 11.3% and 14.8% had depression and anxiety diagnoses respectively using SCID. The overall Cronbach’s alpha for the DASS-21 scale was 0.74. The DASS-21 subscales had Cronbach’s alpha values of 0.66, 0.29 and 0.52 for depression (DASS-D), anxiety (DASS-A) and stress (DASS-S), respectively. The ordinal alpha for DASS-D, DASS-A and DASS-S subscales were 0.83, 0.74 and 0.87, respectively. The area under the ROC curve was 0.76 (95% CI: 0.61; 0.91) for DASS-D and 0.65 for DASS-A. At a cut-off point of one or more, the sensitivity and specificity for DASS-D were 69.2% and 75.5%, whilst DASS-A was 52.9% and 75.5%, respectively. Pearson correlation coefficient for the association between DASS-D and EPDS was r = 0.61, *p* < 0.001.

**Conclusion:**

The DASS-21 had good internal reliability (Cronbach’s alpha), and its ordinal alpha demonstrated good internal reliability for all its sub-scales. Regarding the criterion validation, only the DASS-D and EPDS demonstrated a satisfactory ability to discriminate cases from non-cases. Our findings suggest that health practitioners can use DASS-D as an alternative tool in screening depression as it has fewer questions than EPDS.

**Supplementary Information:**

The online version contains supplementary material available at 10.1186/s12888-022-03994-0.

## Background

Maternal mental health problems remain a global public health challenge. Approximately 13% of women who have given birth in high-income countries suffer from common mental disorder (CMD), primarily depression and/or anxiety [1]. The situation is worse in low and lower-middle-income countries (LMICs), where one in five (19.8%) women who have recently given birth experience a CMD [1]. Untreated CMDs reduce maternal level of functioning [2] and may consequently impair mother-infant interaction [3] and infant cognitive development [4].

Currently, there is a low detection and treatment rate of CMDs in LMICs. There are several reasons for this. First, perinatal mental health for women living in LMICs is only an emerging area of interest for researchers and policymakers. The international agenda has moved beyond survival to understand what is needed to enable women (and infants) to thrive [1]. Second, it has been thought that women living in LMICs are protected from experiencing postpartum mental disorders by social and traditional cultural practices during the postpartum period, which has not been substantiated [1]. Third, the low detection and treatment rate of CMDs in LMICs might be attributed to a lack of culturally sensitive screening tools that are simple to use. Lastly but importantly, in most LMICs, perinatal mental health screening and treatment are yet to be integrated into a routine healthcare package of mother–child dyadic interventions offered when a mother attends well-baby clinics [5].

Several perinatal CMD screening tools such as Edinburgh Postnatal Depression Scale (EPDS) [6], WHO Self-Reporting Questionnaire [7], Patient Health Questionnaire-9 [8], and Post-Traumatic Stress Scale [9] have been validated for screening perinatal mental disorders in Malawi. However, these individual tools are not designed to screen for multiple separate common mental disorders (e.g., depression, anxiety, PTSD). Having a screening tool that is free to use, short in assessment time, and used to screen for more than one CMD is more appealing in a resource-constrained setting. This study aimed to assess the reliability and validity of the Depression, Anxiety, and Stress Scale-21 items (DASS-21) for screening common mental disorders among postpartum women in Malawi. Specifically, we aimed to 1) translate the DASS-21 into Chichewa language and adapt it for use in Malawi, 2) in postnatal Malawian women, establish the reliability of DASS-21 and its subscales and the criterion validity of the adapted DASS-21 against the gold standard of the Diagnostic and Statistical Manual of Mental Disorders, 4^th^ edition (DSM-IV) diagnosis of major or minor depression and generalized anxiety disorder, and 3) compare the test characteristics of the DASS-21 depression scale and EPDS.

## Methods

We conducted a criterion validation study, comparing the responses to the Chichewa language translated and adapted DASS-21 instrument (and the EPDS) against a blinded independent administration of the Structured Clinical Interview for DSM-IV (SCID).

### Participants

Participants were women who had recently given birth in Zomba district, southern Malawi, who participated in the Randomised controlled trial of the Effect of intravenous iron on Anaemia in Malawian Pregnant women (REVAMP – ACTRN12618001268235) trial. The REVAMP study randomized 862 anaemic (haemoglobin level between 5 g/dl and 10 g/dl) pregnant women in their second trimester to either standard treatment of oral ferrous sulphate (200 mg twice a day for 90 days or until birth, whichever came first) or intravenous ferric carboxymaltose (20mgs/kg or maximum of 1000 mg for ≥ 50kgs) once for the whole duration of pregnancy. We included a sub-set of participants who came for follow-up visits with babies aged between 1 and 12 months.

### Measures

#### Depression anxiety stress scale (DASS-21)

The DASS-21 is a widely used screening tool, which can separately measure depression, anxiety, and stress symptoms. The DASS-21 has been derived from the original 42-items DASS developed by Lovibond et al. (1995), which has three sub-scales, namely the depression subscale (DASS-D), Anxiety subscale (DASS-A) and stress subscale (DASS-S) [10]. The DASS-D measures an individual’s hopelessness, positive affect and self-esteem. The DASS-A measures autonomic arousal, situational anxiety, musculoskeletal symptoms, the perceived experience of anxiety arousal and situation anxiety. Finally, DASS-S measures agitation, tension and negative affect [11]. Each subscale comprises seven items that are scored from 0 (did not apply to me at all) to 3 (applied to me very much, or most of the time) for the week preceding the interview to reflect severity. The total score for each DASS-21 subscale ranges from 0 to 21. The items' scores are added and multiplied by two to obtain the total score that can be compared with the original DASS-42. The DASS-21 has been translated into more than 54 languages (http://www2.psy.unsw.edu.au/groups/dass/translations.htm) but not into Chichewa language, and has demonstrated good internal consistency and validity. However, very few studies have established cut-off points for severity classification, limiting its use in clinical settings [12].

### Edinburgh postnatal depression scale (EPDS)

The EPDS is a ten-item self-report scale that has been widely used to detect clinically significant depressive symptoms experienced by women during the postnatal period. Each question is rated on a scale of 0 to 3, and total scores can range from 0 to 30, with a higher score representing more depressive symptoms. If criteria for culturally sensitive translation are met, EPDS has shown to be a reliable screening tool for depression in LMICs [13]. Some authors suggest that EPDS items number 3, 4 and 5 (EPDS-3A) that assess guilt, anxiety and fear can detect anxiety disorders. However, studies have reported mixed findings on the reliability of EPDS-3A in screening anxiety symptoms in other settings [14–17]. In Malawi, the EPDS has been validated for use among pregnant women, and the interviewer-administered Chichewa-translated EPDS has shown to be a valid tool for screening and detecting episodes of both major and minor depression among antenatal women (AUC = 0.767: 95% CI 0.695–0.839) [6].

### Diagnostic and statistical manual of mental disorders: fourth edition (DSM-IV)

The DSM-IV remains the gold standard tool for diagnosing mental disorders. The Structured Clinical Interview for DSM-IV (SCID) uses categorical classifications to divide mental disorders based on criteria with defining features. It requires an individual with clinical training and experience to administer the SCID. The specific diagnostic criteria included in the SCID are only meant to guide, but a diagnosis is informed by clinical judgment [16]. This study used SCID modules for depression (minor and major) and anxiety (current generalised anxiety disorder) previously translated into Chichewa, adapted and used in Malawi [6].

### Procedures

#### Content validation

The DASS-21 was translated from English into Chichewa (Appendix 1) by professional accredited translators at Malawi Liverpool Wellcome Trust Research Centre in Malawi. Chichewa is the widely used language in most parts of Malawi. After that, it was reviewed by a group of health workers and mental health clinicians for appropriateness of language and cultural idioms. This meeting agreed that DASS-Stress subscale item questions measure different constructs from those in the stress module in DSM-IV, and assessment for criterion validation has therefore not been done for this subscale. The DSM-IV was used instead of the current version of DSM-5 as it was actively in use for diagnosing mental health disorders by psychiatrists in this setting when data for this study was collected. A field trial of the reviewed DASS-21 was conducted with 20 women who were pregnant and contributed information on clarity, comprehensibility, and suggestions of culturally sensitive phrases. The tool was then back-translated by an independent professional translator for verification.

### Construct and criterion validation

#### Sample Size

Assuming that the prevalence of postpartum depression was at 10% [6, 18], a minimum sample of 120 participants (including 12 having clinical symptoms of depression) was adequate to achieve a minimum power of 90% to detect a change in the percentage value of sensitivity of DASS-21 items from 0.50 to 0.90 based on the target significance level of 0.05. This sample size was also adequate to detect a change in the value of specificity from 60 to 80%, which only required a minimum of 50 participants (including five participants with clinical signs of depression). The sample size was calculated based on the prevalence of depression as it is the most common and significant public health with serious clinical impact compared to anxiety [19].

### Questionnaire administration

We used a convenience sampling technique to select 120 participants from the REVAMP trial participants who came for their scheduled visits with babies aged between one and 12 months. A maximum of five consecutive participants were assessed in a day to ensure quality assessments. Data collection was performed between November 2020 and May 2021. Participants were first interviewed by 1) a mental health clinician (TK) who administered the SCID modules for major or minor depression, current and generalised anxiety disorder, and 2) a trained registered nurse (GK) who administered the DASS-21 and EPDS in individual interviewers thereafter. This is usual and best practice in settings with low familiarity with test-taking and where many people have low literacy [13]. The mental health clinician had a degree in clinical medicine (mental health) and over four years’ experience using SCID at a mental hospital. The registered nurse was trained on the use of DASS-21 and EPDS and was actively supervised by EM. The two assessors worked independently and were blinded to each other’s scores.

### Data management and quality assurance

The paper-based SCID administered by the mental health clinician was checked for completeness by the EM before entry in Open Data Kit (ODK). The DASS-21 and EPDS were directly collected using a tablet (ODK), and data were checked for completeness before being uploaded to the server. Data were cleaned and checked for consistency before analysis by EM.

### Statistical analysis

#### Internal reliability

The reliability of any given measurement is the extent to which it is a consistent measure of a concept. Internal reliability was measured by both Cronbach’s alpha (most widely and frequently used reliability index) which reflects the extent to which different subsets of the test items produce similar measures [18], and ordinal alpha [20]. We have reported both overall alpha and instrument (DASS-21) subscale alphas for the Cronbach's alpha. The alpha coefficient of reliability ranges from 0 (all items are entirely independent of one another) to 1 (all items are highly related). Alpha coefficient between 0.70 and 0.80 was regarded as acceptable and less than 0.5 as not acceptable [21].

Further analysis of the internal consistency reliability was done by calculating item-test, item-rest and average inter-item correlations. All of these influence and describes the overall item score reliability. However, it is now known that Cronbach’s alpha underestimates the correlation alpha when data from Likert-type scales are used [20]. The polychoric correlation matrix used in ordinal alpha calculation corrects attenuation caused by the scaling of items in Likert-type scales. It tends to estimate reliability more accurately than Cronbach’s alpha [20, 22–24].

### *Correlation between* DASS-21 subscale of depression and EPDS

We examined the convergent validity of the DASS- Depression subscale by assessing its correlation with EPDS. This scale has been demonstrated to be a valid depression screening measure in Malawi*.* A Pearson’s correlation coefficient (*r*) for the DASS-21 subscale of depression and EPDS was also calculated as the scores in both tools were normally distributed. The r correlation measures the strength of the linear relationship between two continuous variables. The following cut-off 0.1 < [*r*] < 0.3, 0.3 < [*r*] < 0.5 and 0.5 < [*r*] < 0.7 and [*r*] > 0.7 were used to measure the strength of association and interpreted as very weak correlation, weak correlation, moderate correlation and strong correlation respectively [25].

### Criterion validation for DASS-D, DASS-A and EPDS against the SCID

Compared to the DSM-IV structured interviews (SCID) scores, we calculated the sensitivity, specificity, and positive and negative likelihood ratio for DASS-21 and EPDS. A positive likelihood ratio (LR +) tells us how much to increase the probability of the disease if the test is positive, while a Negative likelihood ratio (LR-) tells us how much to decrease the probability of the disease if the test is negative. The larger the LR + (≥ 5), the greater the likelihood of the disease and the smaller the LR- (< 5), the lesser the likelihood of disease. (http://medtrain.chm.msu.edu/ebm/Diagnosis/at_likelihood_ratios.html)*.*

The ability of DASS-21 and EPDS to discriminate cases from non-cases was assessed by receiver operating characteristics (ROC) analysis, and its curve was used to select cut-off points for DASS-21 subscales and EPDS. The area under the ROC curve (AUC) is the best parameter for summarizing a screening tool’s overall discriminative value. A value of ≥ 0.70, 0.80 up to 0.90 and > 0.90 is interpreted as reasonable, good and excellent, respectively [26]. Comparisons were made between the AUCs for the DASS-D and EPDS. The criteria for choosing an optimal cut-off value were to maximise both sensitivity and specificity and to set sensitivity values higher than specificity to detect all potential cases. Youden index (sensitivity + specificity – 1) was used to assess the ability of screening tools to balance sensitivity and specificity, and the results were presented as a percentage. It has been recommended that for a test to be useful, the Youden Index should be ≥ 50% [27]. Data analysis was conducted using Stata Version 15.1 (StataCorp LP, College Station, Texas, United States of America, 2017).

### Ethical considerations

This study was nested within the REVAMP trial, approved by the College of Medicine Research and Ethics Committee, Malawi (P.02/18/2357) and the Walter and Eliza Hall Institute of Medical Research Ethics Committee, Australia (WEHI REC 18/02). All participants were given information about the study and either signed a consent form or printed their thumb print (as witnessed by an impartial observer) for those who could not write. Assessments were done in closed rooms to maintain privacy. Participants were given unique identifying numbers, and forms containing their names were kept separately from their files. Participants identified needing further assessment and management were referred to the local Ministry of Health psychiatric services.

## Results

### Descriptive analysis

Overall, 115/120 (95.8%) of the required sample provided complete responses to all the three questionnaires (SCID, DASS-21 and EPDS) administered. The mean age of the included participants was 24 (standard deviation: 6.8) years. 82% of the participants were either married or living together with their husbands, and 68.7% were Christians (Table 1). Most of the participants had either attended primary (60.9%) or secondary (34.8%) school and depended on either casual labour (35.7%) or farming (36.5%) to earn a living.

**Table 1:** Characteristics of the study participants (*N* = 115)

Age (in years) *	*n* (%)
< 20	37 (32.2)
20—29	55 (47.8)
30 – 39	30 – 39
≥ 40	4 (3.5)
Marital status	
Never married	19 (16.6)
Married/Living together	94 (81.7)
Divorced/Separated	2 (1.7)
Education level	
Not attended school	3 (2.6)
Primary	70 (60.9)
Secondary	40 (34.8)
Tertiary	2 (1.7)
Religion	
Christianity	79 (68.7)
Islam	36 (31.3)
Source of income	
Casual work	41 (35.7)
Farming	42 (36.5)
Permanent Employment	32 (27.8)
Tribe	
Chewa	16 (13.9)
Yao	43 (37.4)
Lomwe	33 (28.7)
Mang’anja	5 (4.3)
Tonga	14 (12.2)
Others	4 (3.5)

Age* mean: 24, standard deviation (SD): 6.8

Using the SCID interviews, 13/115 (11.3%) participants were diagnosed with current depression (3 with major depression and 10 with minor depression) and 17/115 (14.8%) were diagnosed with current generalised anxiety disorder. The 3 participants diagnosed with major depression were referred to local psychiatric services for further management.

### Internal reliability of DASS-21 items and EPDS

The overall DASS-21 item test scale reliability results are shown in Table 2. The overall Cronbach’s alpha for DASS-21 was 0.74. The Cronbach’s alpha for the DASS-D subscale, DASS-A subscale and DASS-S subscales were 0.66, 0.29 and 0.52, respectively. Detailed results on item-test correlation, item-rest correlation and average inter-item correlation, which influences the overall Cronbach’s alpha, are shown in Table 2. The internal reliability (Cronbach’s alpha) of EPDS was 0.74, with item-test correlation and item-specific alpha ranging from 0.30 to 0.81 and 0.67 to 0.76, respectively. The ordinal alphas for DASS-D, DASS-A and DASS-S subscales were 0.83, 0.74 and 0.87, respectively.

**Table 2:** DASS-21 Item test scale reliability

Item question	Obs	Sign	Item-test correlation	Item-rest correlation	Average inter-item correlation	Alpha
DASS-Depression subscale						
I found it difficult to work up the initiative to do things	115	+	0.5908	0.3907	0.2151	0.6219
I couldn’t seem to experience any positive feeling at all	115	+	0.4552	0.2263	0.2515	0.6685
I felt that I had nothing to look forward to	115	+	0.6124	0.4183	0.2093	0.6137
I felt down-hearted and blue	115	+	0.5742	0.3697	0.2196	0.6280
I was unable to become enthusiastic about anything	115	+	0.4266	0.1934	0.2592	0.6774
I felt I wasn’t worth much as a person	115	+	0.7136	0.5532	0.1822	0.5720
I felt that life was meaningless	115	+	0.6536	0.4720	0.1983	0.5974
Test Scale Reliability (Cronbach’s alpha)					**0.2193**	**0.6629**
DASS-Anxiety subscale						
I was aware of dryness of my mouth	115	+	0.4242	0.1068	0.0587	0.2722
I experienced breathing difficulty	115	+	0.4748	0.1657	0.0484	0.2336
I experienced trembling (e.g. in the hands)	115	-	0.3392	0.0130	0.0760	0.3305
I was worried about situations in which I might panic and make a fool of myself	115	+	0.4485	0.1347	0.0537	0.2541
I felt I was close to panic	115	-	0.4349	0.1190	0.0565	0.2643
I was aware of the action of my heart in the absence of physical exertion	115	+	0.5410	0.2467	0.0349	0.1781
I felt scared without any good reason	115	-	0.3961	0.0751	0.0644	0.2923
Test Scale Reliability (Cronbach’s alpha)					0.0561	0.2937
DASS-Stress subscale						
I found it hard to wind down	115	+	0.6744	0.4705	0.0963	0.3899
I tended to over-react to situations	115	+	0.2857	0.0052	0.1886	0.5824
I felt that I was using a lot of nervous energy	115	+	0.3669	0.0923	0.1693	0.5501
I found myself getting agitated	115	+	0.6929	0.4963	0.0919	0.3777
I found it difficult to relax	115	+	0.5571	0.3158	0.1241	0.4595
I was intolerant of anything that kept me from getting on with what I was doing	115	+	0.3083	0.0291	0.1832	0.5737
I felt that I was rather touchy	115	+	0.6929	0.4963	0.0919	0.3777
Test Scale Reliability (Cronbach’s alpha)					0.1355	0.5233

### Correlation between DASS-D subscale and EPDS

A Pearson correlation coefficient examined the relationship between scores on the DASS-D subscale and EPDS. The results showed a statistically significant positive and moderate association (*r* = 0.61, *p* < 0.001).

#### Criterion validity for DASS-21, DASS-D, DASS-A and EPDS against SCID

**Fig. 1:**
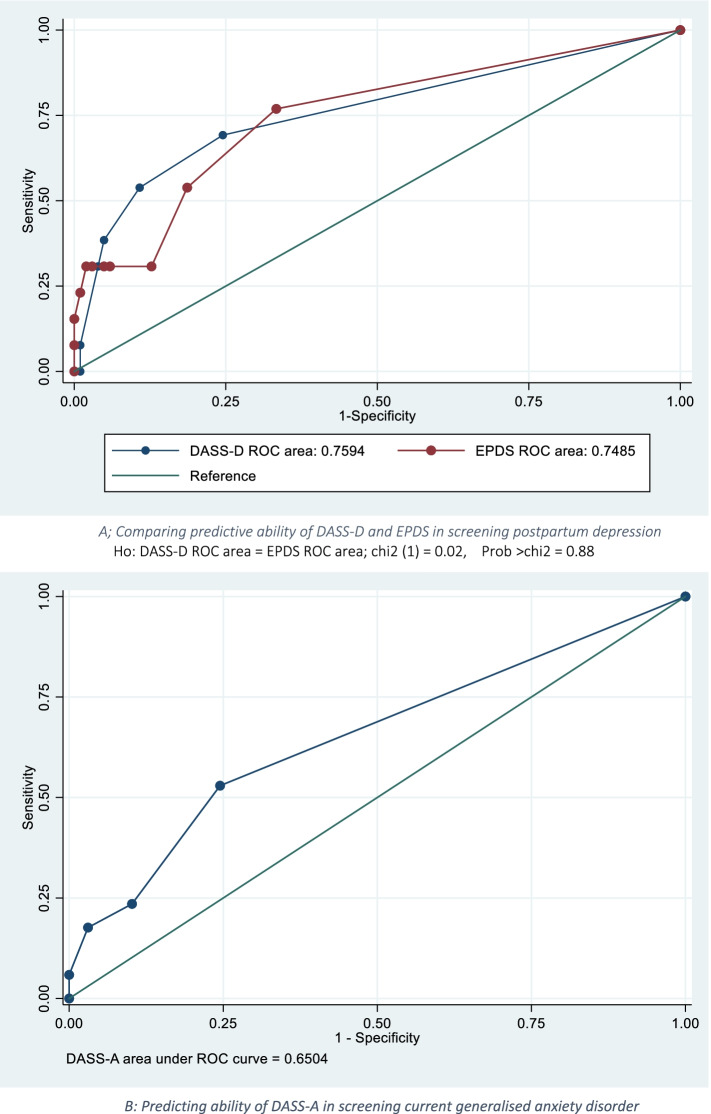
**A** Comparing predictive ability of DASS-D and EPDS in screening postpartum depression Ho: DASS-D ROC area = EPDS ROC area; chi2 (1) = 0.02, Prob > chi2 = 0.8844. **B** Predicting ability of DASS-A in screening current generalised anxiety disorder

The results of sensitivity, specificity, Youden index, cases correctly classified, positive likelihood ratio (LR +) and negative likelihood ratio (LR-) for both DASS-21 (Depression and Anxiety sub-scales) and EPDS compared to SCID are shown in Table 3. The receiver operating curve (ROC) analysis for the DASS-21 depression subscale against the criterion of DSM-IV current depressive episode (minor or major) gave an area under the curve (AUC) value of 0.76 (95% CI: 0.61; 0.91), and details are shown in Fig. 1A. The ROC analysis for the DASS-21 anxiety subscale against the DSM-IV current generalized anxiety disorder criterion gave an AUC value of 0.6505 (95% CI: 0.52; 0.79), as shown in Fig. 1B. The ROC analysis for EPDS against the DSM-IV current depressive episode (minor or major) gave an AUC value of 0.75 (95% CI: 0.60; 0.89). As shown in Fig. 1A, although the AUC for DASS-depression subscale is larger than that of EPDS, the chi-squared test yielded a probability of 0.88, suggesting no significant difference between the two areas. The ROC area for the whole DASS-21 against SCID depression and anxiety diagnoses were 0.71 (95% CI: 0.54; 0.87) and 0.77 (95% CI: 0.62; 0.92) respectively.

**Table 3:** Operating characteristics of DASS-D, DASS-A and EPDS at various cut-off scores for identifying depression and anxiety against the SCID

Cut-point	Sensitivity	Specificity	Youden Index	Correctly Classified	LR +	LR-
DASS-D (Depression subscale)						
> = 0	100.00%	0.00%	0%	11.30%	1.0000	
> = 1	69.23%	75.49%	44.72%	74.78%	2.8246	0.4076
> = 2	53.85%	89.22%	43.07%	85.22%	4.9930	0.5173
> = 3	38.46%	95.10%	33.56%	88.70%	7.8462	0.6471
> = 4	30.77%	96.08%	26.85%	88.70%	7.8462	0.7206
> = 6	7.69%	99.02%	6.71%	88.7§0%	7.8462	0.9322
> = 7	0.00%	99.02%	-0.98%	87.83%	0.0000	1.0099
> = 8	0.00%	100.00%	0%	88.70%		1.0000
DASS-A (Anxiety subscale)						
> = 0	100.00%	0.00%	0%	14.78%	1.0000	
> = 1	52.94%	75.51%	28.45%	72.17%	2.1618	0.6232
> = 2	23.53%	89.80%	13.33%	80.00%	2.3059	0.8516
> = 3	17.65%	96.94%	14.59%	85.22%	5.7647	0.8495
> = 5	5.88%	100.00%	5.88%	86.09%		0.9412
> = 6	0.00%	100.00%	0%	85.22%		1.0000
Edinburgh Postpartum Depression Scale (EPDS)						
> = 0	100.00%	0.00%	0%	11.30%	1.0000	
> = 1	76.92%	66.67%	43.59%	67.83%	2.3077	0.3462
> = 2	53.85%	81.37%	35.22%	78.26%	2.8907	0.5672
> = 3	30.77%	87.25%	18.02%	80.87%	2.4142	0.7934
> = 4	30.77%	94.12%	24.89%	86.96%	5.2308	0.7356
> = 5	30.77%	95.10%	25.87%	87.83%	6.2769	0.7280
> = 6	30.77%	97.06%	27.83%	89.57%	10.4615	0.7133
> = 7	30.77%	98.04%	28.81%	90.43%	15.6923	0.7062
> = 8	23.08%	99.02%	22.10%	90.43%	23.5385	0.7768
> = 9	15.38%	100.00%	15.38%	90.43%		0.8462
> = 12	7.69%	100.00%	7.69%	89.57%		0.9231
> = 13	0.00%	100.00%	0%	88.70%		1.0000

*LR +* Likelihood Ratio Positive, *SCID* Structured Clinical Interviews for Diagnostic Statistical Manual, *LR-* Likelihood Ratio Negative

### Choice of cut-off points

A cut-off point of 1 or higher on the DASS-D depression subscale provided the best combination of sensitivity and specificity (optimal point) in detecting a minor or major depression diagnosis. This cut-off point gave 69.2% and 75.5% for sensitivity and specificity, respectively (Youden Index: 44.7%). At this cut-off point (1 or higher), the correct classification was 74.8%, with a positive likelihood ratio of 2.8 and a negative likelihood ratio of 0.4 (Table 3). For the DASS-A (anxiety subscale), the optimal cut-off point was obtained at a cut-off point of 1 or higher, which gave a sensitivity of 52.9% and specificity of 75.5% (Youden Index: 28.4%). This cut-off point gave a correct classification value of 72.2% and 2.16 and 0.62 for positive and negative likelihood ratios, respectively (Table 3). For EPDS, the best combination for sensitivity and specificity was 76.9% and 66.7% (Youden Index: 43.6%), respectively, at a cut-off point of 1 or higher. This cut-off point gave a correct classification value of 72.2%, with a positive likelihood ratio value of 2.16 and a negative likelihood ratio of 0.62 (Table 3).

## Discussion

### Reliability and validity of DASS-21

This study aimed to determine the reliability and validity of DASS-21 in screening for common mental disorders (CMDs) among postpartum women in Malawi. We found that DASS-21 as an overall tool has a good internal reliability. Inconsistent findings were observed between Cronbach’s alpha and Ordinal alpha values for the DASS-21 subscales with the later indicating good internal reliability for all DASS-21 subscales. The internal reliability of the scale is defined as the ability of the scale to measure the construct in question without the influence of measurement error from the use of different questions. A high internal reliability scale has items that are consistent enough whilst a low internal reliability scale has items that are different from one another creating a larger error component [22]. Only the DASS-D and EPDS demonstrated a satisfactory ability to discriminate participants with depression from non-depressed.

Using Cronbach’s alpha values, our findings indicated that the internal consistency of the whole DASS-21 was good (Cronbach’s alpha = 0.74). However, internal consistency was unsatisfactory for the DASS-21 depression and stress subscale and very poor for the DASS-21 anxiety subscales. These findings are not consistent with those reported in high-income countries where the Cronbach’s alpha coefficient for the DASS-21 subscales ranged from good to excellent, indicating good internal consistency [25–27]. A study in Nigeria also reported high Cronbach’s alpha for all DASS-21 subscales [28]. Evidence suggests that increasing the response options in a Likert scale question provides more accurate composite score estimates with sampling variability, consequently increasing item test reliability [23]. However, comprehending and choosing the right response option depends on individual literacy levels. Differences might influence the above discrepancies in findings in participants’ level of education among studies. For example, most of the participants in our study had received primary school education compared to a study in Nigeria which involved medical students. In high-income countries, most of the study participants had a minimum of a first degree. Vignola and Tucci (2014) suggested that participants with low literacy might have difficulties understanding and rating subjective emotional states presented on a Likert scale [29]. Stewart et al. (2009) addressed the challenges of Likert scale rating among low literate participants by introducing pictorial faces that demonstrate variation in an emotional state [6]. However, these additions might add complexity to tool use, requiring adequate training before use and raising feasibility constraints, especially in LMICs where resources are limited.

It is known that DASS-21 anxiety subscale items measure two different constructs, namely somatic symptoms and negative emotional symptoms. Additionally, it has been suggested that women with common mental disorders in LMICs tend to endorse more somatic symptoms than negative emotions [30]. This was evident in this study as three of the DASS-21 anxiety subscale items indicated negative inter-item correlation (Table 2). This consequently reduced the average inter-item correlation as items that are not related and going in different directions end up cancelling each other, thereby affecting the overall Cronbach alpha (Cronbach alpha coefficient for DASS-A subscale = 0.29).

Using ordinal alpha which of recent has been recommended as an alternative for Cronbach’s alpha for Likert scales [20], high reliability values for all DASS-21 subscales were reported. A detailed explanation on the pros and cons of ordinal alpha vs Cronbach’s alpha are beyond the scope of this paper and reported elsewhere [20, 23, 24]. However, it is important to note that two assumptions are associated with Cronbach’s alpha, namely 1) the tau-equivalence (all items are of equal importance when measuring the unobserved construct, but that the respective error variances for each item are allowed to differ) and 2) uncorrelated errors [20]. When using a Likert scale, correlated errors might be introduced through 1) not creating enough categories to fully represent the construct in question, 2) when the underlying continuous distribution does not match the observed categorical distribution of scores in terms of skew and 3) misclassification errors. These affect Cronbach’s alpha as the Pearson product-moment correlations are attenuated when using Likert scale data [20].

A large difference was observed in the Cronbach’s alpha compared to the Ordinal alpha for the DASS-21 anxiety subscale (Cronbach’s alpha = 0.29 versus Ordinal alpha = 0.74). This difference was expected as items that are not measuring the same construct as in the DASS-anxiety subscale tend to yield lower values of Cronbach’s alpha as the Pearson covariance matrix is substantively distorted [20, 22]. The high ordinal alpha value, in this case, is a result of the polychoric correlation matrix, which corrects for the attenuation caused by the transformation of categorical Likert scale data into unobserved continuous data [20, 22].

Another important finding in this study is that none of the DASS-21 item subscales combined sufficiently to give high sensitivity and specificity for routine screening for common mental disorders even at a cut-off point of 1 or more. The Youden indexes for all DASS-21 subscales and EPDS are below 50%, demonstrating a lack of the diagnostic tools to detect either disease or health. However, it is important to note that even the current EPDS that was validated in this setting and reported to show good test characteristics as a screening measure for depressive disorders had Youden Index < 50% (calculated: 48.9%, not shown in the article) [6]. Perhaps this emphasizes that screening tools are not for diagnosing diseases but rather for identifying probable cases that can be referred for further assessments. Additionally, the prevalence of depression in this setting was very low (with only 3 participants diagnosed with major depression), which may affect the sensitivity and specificity of the screening tools [31, 32].

Similar findings were also reported by Tran et al. [11] in the validation of the DASS-21 as a screening instrument for depression and anxiety in a rural community-based cohort of northern Vietnamese women. Using exploratory factor analysis, Tran et al. [11] found DASS-21 not good at distinguishing different conditions. Still, the single factor in which all subscales are added had some value in detecting any CMD [30]. Hanlon et al. (2015) argued that in low-literacy settings, it is possible that the potential accuracy of a screening tool can be offset by the complexity of the multiple response categories presented in Likert scales [33]. The area under the ROC curve for the DASS-21 depression subscale was 0.76, and EPDS was 0.75; this is a moderately high value suggesting the reasonable diagnostic ability of both the DASS depression subscale and EPDS. The AUC for EPDS in this study is almost similar to what was reported (0.77: 95% CI; 0.695–0.839) in a previous study that validated the use of EPDS in screening minor or major depression among antenatal women in our setting [6]. A comparison of AUCs between EPDS (an established tool in our setting for screening postpartum depression) and the DASS-D subscale indicated no significant difference between the two areas. Our results have also shown a statistically significant positive and moderate association between the DASS-21 depression subscale and EPDS score, suggesting a DASS-21 depression subscale is a reliable tool for screening minor or major depression.

### Strength and limitation

This study is the first study in Malawi to conduct criterion validation of the DASS-21 subscales against SCID diagnoses. Besides using Cronbach’s alpha which is a standard analysis of internal reliability (item inter-relatedness) of psychometric tool, ordinal alpha was also reported. However, we acknowledged that we did not do test–retest reliability due to resource constraints. Our study findings may have reduced generalisability. We only included mothers who had babies aged between one and 12 months old who were previously enrolled in a randomisation study that recruited only anaemic pregnant women. Lastly, most of the participants in this study received primary education, and the findings should not be generalised to a population with a high literacy level.

## Conclusion

The study found DASS-21 as an overall item having good internal reliability. Still, none of its subscales (DASS-A and DASS-S) had satisfactory internal reliability except for the depression subscale, as indicated by Cronbach’s alpha. However, ordinal alpha demonstrated good internal reliability for all DASS-21 subscales. Researchers have now agreed that ordinal alpha may be the most appropriate measure of internal reliability for Likert-type data. Our findings suggest that DASS-D, DASS-A, and DASS-S subscales have good internal reliability for screening common mental disorders in Malawi. A positive and statistically significant moderate correlation between DASS-D subscale and the EPDS was also observed.

Regarding the criterion validation, only the DASS-depression subscale and EPDS demonstrated satisfactory ability to discriminate cases from non-cases with no significant difference between their ability to detect either minor or moderate depression among women who have recently given birth. Therefore, health practitioners in this setting have a choice of whether to use the DASS-D subscale or EPDS for screening minor/major depression based on their preferences. We recommend future studies consider including uncomplicated strategies to maximise the use of the DASS-21 Likert response option in the low literacy population.

## Supplementary Information

**Additional File 1.** 

## Data Availability

The datasets generated and/or analysed during the current study are not publicly available. The main study (REVAMP trial) is still ongoing but is available from the corresponding author upon reasonable request.

## Abbreviations

CMDs: Common Mental Disorders
DASS-21: Depression Anxiety Stress Scale-21 items
EPDS: Edinburgh Postpartum Depression Scale
GK: Gladys Kusiwa
LMICs: Lower and Middle-Income Countries
ROC: Receiver Operating Characteristics
SCID: Structured Clinical Interview for DSM-IV
TK: Twisile Kalinga
