# Supplementary material for: Reliability and validity of depression anxiety stress scale (DASS)-21 in screening for common mental disorders among postpartum women in Malawi
Source: BMC Psychiatry. 2022 May 24;22:352. doi: 10.1186/s12888-022-03994-0 (PMC9128196; doi:10.1186/s12888-022-03994-0)
Supplement: Supplementary file 1 — Additional File 1. [file 12888_2022_3994_MOESM1_ESM.docx]

**DEPRESSION ANXIETY STRESS SCALE (DASS-21) CHICHEWA VERSION**

| **Zoyenera Kusata:** Chonde sonyezani kuti chiganizo chili m’munsimu chinagwira bwanji ntchito pa inu mu sabata yapitayi. Palibe mayankho 'olondola' kapena 'olakwika'. Musatenge nthawi yambiri pa chiganizo china chilichonse | | |
| --- | --- | --- |
| 01. | Zimandivuta kubwerera m’chimake | (0) Sizinandichitikire oro ndi pang’ono pomwe  (1) Zimandichitikira nthawi zina oro kuti mwaapo ndi apo  (2) Zimandichitikira kwambiri panthawi zina  (3) Zimandichitikira kwambiri kwa nthawi zambiri |
| 02. | Ndimadziwa za kuuma kwa mkamwa mwanga | (0) Sizinandichitikire oro ndi pang’ono pomwe  (1) Zimandichitikira nthawi zina oro kuti mwa apo ndi apo  (2) Zimandichitikira kwambiri panthawi zina  (3) Zimandichitikira kwambiri kwa nthawi zambiri |
| 03. | Zikuonetsa kuti sindimatha kumva bwino | (0) Sizinandichitikire oro ndi pang’ono pomwe  (1) Zimandichitikira nthawi zina oro kuti mwa apo ndi apo  (2) Zimandichitikira kwambiri panthawi zina  (3) Zimandichitikira kwambiri kwa nthawi zambiri |
| 04. | Ndinkakhala ndibvuto m’mapumidwe (monga: Kupuma mothamanga, Kubanika ngakhale ndisakugwira ntchito yolemetsa) | (0) Sizinandichitikire oro ndi pang’ono pomwe  (1) Zimandichitikira nthawi zina oro kuti mwa apo ndi apo  (2) Zimandichitikira kwambiri panthawi zina  (3) Zimandichitikira kwambiri kwa nthawi zambiri |
| 05. | Ndimakhala ndi ulesi pofuna kugwira ntchito | (0) Sizinandichitikire oro ndi pang’ono pomwe  (1) Zimandichitikira nthawi zina oro kuti mwa apo ndi apo  (2) Zimandichitikira kwambiri panthawi zina  (3) Zimandichitikira kwambiri kwa nthawi zambiri |
| 06. | Ndinali ndi chizolowezi chochita zinthu ndi mkwiyo | (0) Sizinandichitikire oro ndi pang’ono pomwe  (1) Zimandichitikira nthawi zina oro kuti mwa apo ndi apo  (2) Zimandichitikira kwambiri panthawi zina  (3) Zimandichitikira kwambiri kwa nthawi zambiri |
| 07. | Ndimanjenjemera (mwachisazo: manja) | (0) Sizinandichitikire oro ndi pang’ono pomwe  (1) Zimandichitikira nthawi zina oro kuti mwa apo ndi apo  (2) Zimandichitikira kwambiri panthawi zina  (3) Zimandichitikira kwambiri kwa nthawi zambiri |
| 08. | Ndimazimva kuti ndimagwiritsa ntchito kwambiri ubongo kapena kuziva kuti ubongo wanu unali wotopa | (0) Sizinandichitikire oro ndi pang’ono pomwe  (1) Zimandichitikira nthawi zina oro kuti mwa apo ndi apo  (2) Zimandichitikira kwambiri panthawi zina  (3) Zimandichitikira kwambiri kwa nthawi zambiri |
| 09. | Ndimadandaula ndi nyengo zochititsa mantha zimene zimkandionetsa ngati ndine chitsiru | (0) Sizinandichitikire oro ndi pang’ono pomwe  (1) Zimandichitikira nthawi zina oro kuti mwa apo ndi apo  (2) Zimandichitikira kwambiri panthawi zina  (3) Zimandichitikira kwambiri kwa nthawi zambiri |
| 10. | Ndimazimva kupanda chiyembekezo | (0) Sizinandichitikire oro ndi pang’ono pomwe  (1) Zimandichitikira nthawi zina oro kuti mwa apo ndi apo  (2) Zimandichitikira kwambiri panthawi zina  (3) Zimandichitikira kwambiri kwa nthawi zambiri |
| 11. | Ndimazimva kubalalika | (0) Sizinandichitikire oro ndi pang’ono pomwe  (1) Zimandichitikira nthawi zina oro kuti mwa apo ndi apo  (2) Zimandichitikira kwambiri panthawi zina  (3) Zimandichitikira kwambiri kwa nthawi zambiri |
| 12. | Zimandivuta kudekha/kukhala bata | (0) Sizinandichitikire oro ndi pang’ono pomwe  (1) Zimandichitikira nthawi zina oro kuti mwa apo ndi apo  (2) Zimandichitikira kwambiri panthawi zina  (3) Zimandichitikira kwambiri kwa nthawi zambiri |
| 13. | Ndimazimva kudandaula komanso kukhumudwa | (0) Sizinandichitikire oro ndi pang’ono pomwe  (1) Zimandichitikira nthawi zina oro kuti mwa apo ndi apo  (2) Zimandichitikira kwambiri panthawi zina  (3) Zimandichitikira kwambiri kwa nthawi zambiri |
| 14. | Palibe chimene chimandikhudza pa china chilichonse chimene chimandiletsa kupitiliza kuchita zimene ndimachita | (0) Sizinandichitikire oro ndi pang’ono pomwe  (1) Zimandichitikira nthawi zina oro kuti mwa apo ndi apo  (2) Zimandichitikira kwambiri panthawi zina  (3) Zimandichitikira kwambiri kwa nthawi zambiri |
| 15. | Ndimazimva kukhala ndi mantha | (0) Sizinandichitikire oro ndi pang’ono pomwe  (1) Zimandichitikira nthawi zina oro kuti mwa apo ndi apo  (2) Zimandichitikira kwambiri panthawi zina  (3) Zimandichitikira kwambiri kwa nthawi zambiri |
| 16. | Ndinalibe chikhumbo-khumbo pa china chilichonse | (0) Sizinandichitikire oro ndi pang’ono pomwe  (1) Zimandichitikira nthawi zina oro kuti mwa apo ndi apo  (2) Zimandichitikira kwambiri panthawi zina  (3) Zimandichitikira kwambiri kwa nthawi zambiri |
| 17. | Ndimazimva kuti ndine munthu osayenera | (0) Sizinandichitikire oro ndi pang’ono pomwe  (1) Zimandichitikira nthawi zina oro kuti mwa apo ndi apo  (2) Zimandichitikira kwambiri panthawi zina  (3) Zimandichitikira kwambiri kwa nthawi zambiri |
| 18. | Ndimazimva kukhala wokwiya-kwiya | (0) Sizinandichitikire oro ndi pang’ono pomwe  (1) Zimandichitikira nthawi zina oro kuti mwa apo ndi apo  (2) Zimandichitikira kwambiri panthawi zina  (3) Zimandichitikira kwambiri kwa nthawi zambiri |
| 19. | Ndimazindikira zimene mtima wanga umachita ngakhale ndisakuchita masewela olimbitsa thupi (e.g. kumva kukwera kwa mulingo wa kagundidwe ka mtima, mtima kusagunda kamphindi kena kake) | (0) Sizinandichitikire oro ndi pang’ono pomwe  (1) Zimandichitikira nthawi zina oro kuti mwa apo ndi apo  (2) Zimandichitikira kwambiri panthawi zina  (3) Zimandichitikira kwambiri kwa nthawi zambiri |
| 20. | Ndimachita mantha popanda chifukwa chodziwika bwino | (0) Sizinandichitikire oro ndi pang’ono pomwe  (1) Zimandichitikira nthawi zina oro kuti mwa apo ndi apo  (2) Zimandichitikira kwambiri panthawi zina  (3) Zimandichitikira kwambiri kwa nthawi zambiri |
| 21. | Ndimazimva kuti moyo ulibe tanthauzo | (0) Sizinandichitikire oro ndi pang’ono pomwe  (1) Zimandichitikira nthawi zina oro kuti mwa apo ndi apo  (2) Zimandichitikira kwambiri panthawi zina  (3) Zimandichitikira kwambiri kwa nthawi zambiri |

**STAFF ID**: ______________________________
